# Virginia State Dental Association.—Second Annual Meeting

**Published:** 1872-01

**Authors:** James B. Hodgkin


### ARTICLE III.

### *Virginia State Dental Association.—Second Annual Meeting.*

REPORTED BY JAMES B. HODGKIN, D. D. S.

The second annual meeting of the Virginia State Dental Association was held in Richmond on the 2nd and 3rd of November 1871, at the office of Dr. Judson B. Wood. While the attendance was not as large as was hoped for, yet the spirit of desire for dental improvement was so fully manifested, and the enthusiasm expressed so great, that those present were more than ever convinced of the importance as well as the utility of organization, and of the fact also that the Society, though only in its second year, was a decided success.

The meeting was called to order by the President, Dr. James F. Thompson, and after usual preliminary business, the address of the President was read. It is published elsewhere. The profession was represented by delegates from Richmond, Fredericksburg, Norfolk, Staunton, Alexandria and other places. Quite a number of new members were received, and a more perfect organization of the Society effected.

The chairman of the executive committee, Dr. H. M. Grant, reported that he had corresponded with some three hundred and twenty-five dentists in the State, with a view to securing their interest in the organization. Many had responded. Among these responses was the following interesting letter from Dr. W. W. H. Thackston:

FARMVILLE, VA., Oct. 31st, 1871.

*My Dear Doctor:*

I received your kind letter calling my attention to the "Annual meeting of Virginia Society of Dental Surgeons." I should have replied promptly but for sickness at the time, and the hope that I might make a personal acknowledgement at the meeting.

I am greatly grieved and disappointed that circumstances forbid my leaving home at this time; and I now write to let you know, and through you to inform the Association, that it is with the most sincere and unfeigned regret that I am forced to abandon the purpose of being present at the meeting for this year, and of aiding to the extent of my limited resources and ability in the furtherance and accomplishment of the objects and designs of the Society. Feeble as that aid would be, confessedly feeble as my resources are, I

yet cherish the desire to do my personal and individual part in the work of upholding and promoting the advancement of dental science. I feel no relaxation or abatement of interest, as my repeated absence from the meetings of the Society might imply, but a daily growing and strengthening desire for the welfare and improvement of our specialty of the "healing art." More than that, I desire Southern growth and improvement; I desire that Virginia should take her place in the forefront of that column whose march is onward and upward, and whose goal is purity and perfection. I want Virginia talent developed and cultivated, Virginia invention fostered and encouraged, and Virginia resources and achievements brought out and acknowledged until "Virginia Dentist" shall be as proud a title in this country as "American Dentist" now is in the ports and capitals of Europe.

To accomplish so desirable a result there is but one sure, certain and comparatively speedy method, and that method has happily been adopted. Association, *association;* concert and harmonious, persistent, determined *action.* You have taken hold of the great principle that underlies all human triumph, that foreshadows all great achievements. And more than all you have to do, is patiently to work out the details; and in a few short years Virginia Dentistry will be full up and along side of Virginia law, medicine, theology, mathematics, or any other art or science, studied and practiced by human intellect and human hands.

Do me the kindness to present my cordial greeting to the "Virginia Dental Association;" tell them that though not with them in person, I am of them, soul and body, that I sympathize fully and earnestly in their objects and ends, that I will share with them all their labors and burdens, and will rejoice with them in whatever of good their efforts may accomplish.

In great haste,

Very truly, and sincerely yours,

W. W. H. Thackston.

Dr. H. M. Grant was elected President for the ensuing year, and Drs. James F. Thompson, Wm. B. Pleasants, and A. B. Arthur, Vice Presidents. Dr. George F. Keesee was elected Secretary, and Dr. J. Wood, Treasurer, and Drs. Thompson, Scribner, and Mills, the Executive Committee.

Dr. Thompson took occasion in retiring from the chair, very gratefully to thank the members of the Association for the uniform courtesy and kindness he had received from them, and to bespeak for the new presiding officer, the same kindly feelings and treatment.

Dr. Grant took the chair, briefly thanked the Association for the honor conferred on him. He stated that not the least of the benefits arising out of this Association, was the bringing together in harmony and good fellowship, gentlemen from different sections of the State.

Profs. F. J. S. Gorgas, and P. H. Austen of the Baltimore Dental College, Prof. J. Taft, of the Ohio College, and Dr. F. Y. Clark, of Savannah Ga., were elected corresponding members, and Prof. H. R. Noel, of the Baltimore College was elected an honorary member. The code of ethics of the Southern Dental Association was read by Dr. Thompson, and on his motion, adopted as the code by which this Association be governed.

The following appointments of delegates and committees were made:

Dr. J. Hall Moore was appointed a delegate to represent the Association in the next meeting of the Southern Dental Association, and Drs. Wood, Johnston, H. C. Jones, Scribner, and Thompson, delegates to the American Dental Association, to meet at Niagara, N. Y. The President announced the following committees:

*Physiology.*—James Johnston, W. B. Pleasants.

*Dental Pathology and Surgery.*—William A. Mills, J. Hall Moore.

*Histology and Microscopy.*—J. B. Hodgkin, F. A. Jeter.

*Chemistry.*—A. B. Arthur, W. M. Dorsett.

*Dental Therapeutics.*—S. H. Henkel, R. N. Hudson.

*Operative Dentistry.*—J. B. Wood. G. A. Sprinkel.

*Mechanical Dentistry.*—E. R. Perow, J. W. Scribner.

*Dental Literature.*— J. F. Thompson, F. F. Henley.

To deliver the address of welcome to the Southern Dental Association, Dr. W. W. H. Thackston, and Committee of Arrangements for the same meeting, Drs. Wood, Steele, and Thompson.

*Voluntary Essays.*—George F. Keesee, T. M. Henley, George B. Steele.

*Membership.*—J. F. Thompson, J. Hall Moore, J. B. Hodgkin.

Arrangements were made for a meeting of the Association in July, the time when the Southern Dental Association will meet in Richmond, and preparations made for a Virginia welcome to that body.

Dr. Grant read an essay on "Professional Intercourse," which was received with marked attention and greeted with warm applause. It is published elsewhere.

Owing to the infancy of the Association and the newness of its legislative machinery, time was lost in discussion over constitutional and other points, which might perhaps have been devoted to better purposes. It is certain, however, that by the time another annual meeting arrives, with the work laid out for the various committees and essayists, and the discussion of questions arising out of these reports when made, the Association will have abundant material for thought and action. While we would not disparage the claims of the older dentists, and while many of them we revere as fathers and lights in the profession, yet we could not but be gratified at the enthusiasm and ardor of the few young men, who almost unaided have labored and striven successfully to form and place on a solid basis this Association. It is an honor to them and its success is their praise.

The next annual session of the Association will be held in Richmond, in November, 1872.

